# To Members of the Illinois State Medical Society

**Published:** 1854-11

**Authors:** 


### EDITORIAL.

*To Members of the Illinois State Medical Society:*

THE volume of the Transactions of this Society for 1854 has been published and mailed to all who have paid the annual assessment of Two Dollars. The names of all such are published in the list at the end of the volume.

But there are many who have been and are still included as *members* of the Society who have not paid their assessment, and the amount of money received into the treasury is not yet sufficient to pay the expenses of publication. It will be seen from the notice of the volume of Transactions in this number of the journal, that it contains some matter of permanent value.

Will not all those whose names have been enrolled on the list of members of the Society, who have not already done so, forward without delay their names with the annual assessment, and thereby furnish themselves with the Transactions, and the treasury with funds. It will save time and trouble if all applications for copies are addressed directly to N. S. Davis, Treasurer of the Society, Chicago, Ill. D.

---

THE next number, which is the last of the present volume, will contain a leading article on a form of *Jaundice*, which was quite prevalent during the middle part of the last winter, and cases of which are again becoming frequent. With the commencement of the new volume, we shall resume the series of Articles on the Pathology of Fevers, which was commenced in volume ii., and continue them until they embrace a full exposition of our views of the *causes* and *treatment* of this large and important class of diseases.

In the meantime we hope our friends will not withhold their contributions to our columns.

